# Screening of primary open-angle glaucoma diagnostic markers based on immune-related genes and immune infiltration

**DOI:** 10.1186/s12863-022-01072-8

**Published:** 2022-08-24

**Authors:** Lingge Suo, Wanwei Dai, Xuejiao Qin, Guanlin Li, Di Zhang, Tian Cheng, Taikang Yao, Chun Zhang

**Affiliations:** 1 Department of Ophthalmology, Peking University Third Hospital, 49 North Garden Road, Haidian District, Beijing, 100191 PR China; 2 Beijing Key Laboratory of Restoration of Damaged Ocular Nerve, Peking University Third Hospital, 49 North Garden Road, Haidian District, Beijing, 100191 PR China; 3 Department of Ophthalmology, The Second Hospital of Shandong University, Shandong, China; 4 Clinical Stem Cell Research Center, Peking University Third Hospital, Beijing, China; 5 School of Basic Medicine Sciences, Peking University, Beijing, China

**Keywords:** Bioinformatics analysis, Primary open-angle glaucoma, Optic nerve, Trabecular meshwork, Immune infiltration

## Abstract

**Purpose:**

Primary open-angle glaucoma (POAG) continues to be a poorly understood disease. Although there were multiple researches on the identification of POAG biomarkers, few studies systematically revealed the immune-related cells and immune infiltration of POAG. Bioinformatics analyses of optic nerve (ON) and trabecular meshwork (TM) gene expression data were performed to further elucidate the immune-related genes of POAG and identify candidate target genes for treatment.

**Methods:**

We performed a gene analysis of publicly available microarray data, namely, the GSE27276-GPL2507, GSE2378-GPL8300, GSE9944-GPL8300, and GSE9944-GPL571 datasets from the Gene Expression Omnibus database. The obtained datasets were used as input for parallel pathway analyses. Based on random forest and support vector machine (SVM) analysis to screen the key genes, significantly changed pathways were clustered into functional categories, and the results were further investigated. CIBERSORT was used to evaluate the infiltration of immune cells in POAG tissues. A network visualizing the differences between the data in the POAG and normal groups was created. GO and KEGG enrichment analyses were performed using the Metascape database. We divided the differentially expressed mRNAs into upregulated and downregulated groups and predicted the drug targets of the differentially expressed genes through the Connectivity Map (CMap) database.

**Results:**

A total of 49 differentially expressed genes, including 19 downregulated genes and 30 upregulated genes, were detected. Five genes ((Keratin 14) KRT14, (Hemoglobin subunit beta) HBB, (Acyl-CoA Oxidase 2) ACOX2, (Hephaestin) HEPH and Keratin 13 (KRT13)) were significantly changed. The results showed that the expression profiles of drug disturbances, including those for avrainvillamide-analysis-3, cytochalasin-D, NPI-2358, oxymethylone and vinorelbine, were negatively correlated with the expression profiles of disease disturbances. This finding indicated that these drugs may reduce or even reverse the POAG disease state.

**Conclusion:**

This study provides an overview of the processes involved in the molecular pathogenesis of POAG in the ON and TM. The findings provide a new understanding of the molecular mechanism of POAG from the perspective of immunology.

## Background

Glaucoma is the leading cause of irreversible blindness worldwide. With the growing number and proportion of older persons in the population, it is projected that 111.8 million people will have glaucoma in 2040 [1]. Primary open-angle glaucoma (POAG) is the most common type of glaucoma, accounting for 60–70% of all glaucoma patients [2]. In POAG, the anterior and posterior segments of the eye are affected, and serious damage may be inflicted upon the trabecular meshwork (TM) and optic nerve (ON) [2–4]. The TM is a specialized eye tissue essential for the regulation of aqueous humor outflow and control of intraocular pressure (IOP), disturbances of which may lead to elevated IOP and glaucoma [5]. In general, POAG has an insidious onset and develops painlessly and quietly, visual problems often late in the course of the disease, when significant and irreversible ON damage occurs [1]. Neuroprotective therapies are not available, and current treatments are limited to lowering IOP, which can slow disease progression at early disease stages. However, over 50% of glaucoma cases are not diagnosed until irreversible ON damage has occurred [6].

Numerous POAG patient data have been collected in research [5, 7, 8], but the molecular pathogenesis of POAG remains largely obscure. Therefore, an effective treatment option that addresses these molecular changes is still missing. In recent years, accumulating evidence has shown that immune cell infiltration plays an important role in POAG development [9]. Zhang et al. generalized that POAG may be associated with systemic disorders, mainly those related to the nervous system, endocrine system and immune systems. It has been firmly established that the neuroendocrine system and immune system closely interact through mediators, such as hormones, neuropeptides, neurotransmitters and cytokines [10]. Cytokines mediate the biological effects of the immune system, and our previous study revealed an imbalance of T-helper (Th) 1-derived and Th2-derived cytokines in the serum of patients with glaucoma [11]. We also collected data from irises of normal individuals and those with POAG or chronic angle-closure glaucoma (CACG) [12].

Bioinformatics is an interdisciplinary subject that combines a broad spectrum of domains, including the fields of molecular biology, information science, statistics and computer science [13]. Machine learning, a trendy subfield of artificial intelligence (AI), focuses on extracting and identifying insightful and actionable information from big and complex data using different types of neural networks [14]. It is of great significance to reveal the molecular mechanism of disease by using these emerging technologies. Using omics technologies, we are able to measure the expression of several thousand molecules from one sample of affected tissue, leading to an exponential increase in data [15]. The data were used in bioinformatics analyses to identify key transcription factors (TFs) associated with POAG to examine the pathogenesis of glaucoma and may provide a basis for the diagnosis of glaucoma and drug development. CIBERSORT is a method to describe the composition of immune cells in complex tissues based on their gene expression profiles [16]. Few studies have used CIBERSPORT to analyze immune cell infiltration in POAG. In this study, we identified the key genes from TM tissue and ON tissue in patients with POAG compared with normal controls. The aim of this study was to gain a deeper understanding of the molecular pathogenesis of POAG by applying integrative bioinformatics analysis to the available human gene expression data of the TM and ON tissues in patients with POAG and controls. The obtained results enable us to identify possible drug targets to modulate the disease outcome.

## Results

### Systematic search

**Fig. 1:**
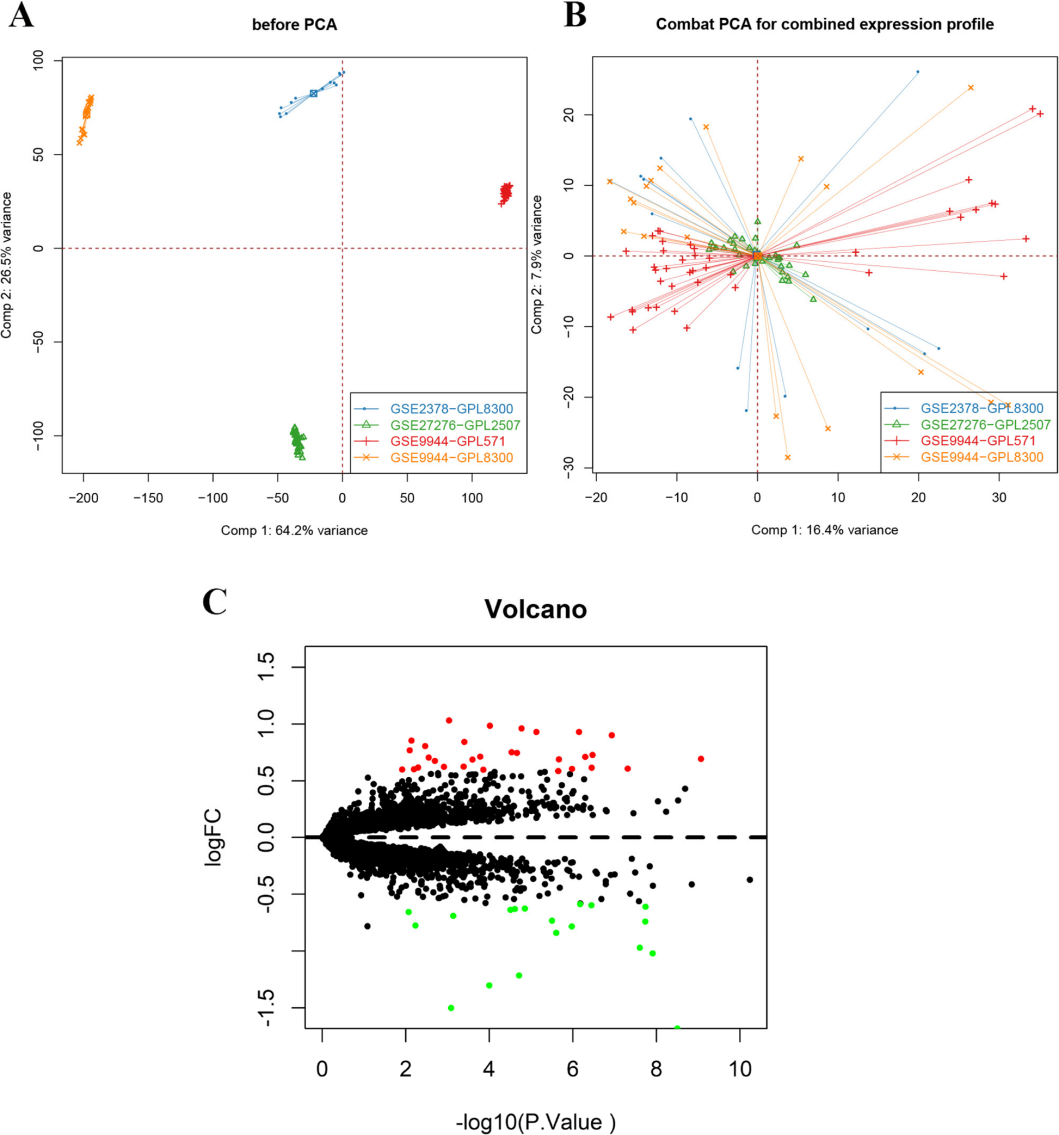
Two-dimensional PCA cluster plot before and after PCA for the combined expression profile. **A, B** shows two-dimensional PCA cluster plots before and after PCA for the combined expression profile. After correcting the batch effect, we combined the four GEO datasets GSE27276, GSE2378, GSE9944 (GPL8300) and GSE9944 (GPL571) into the expression profiles of 110 samples (control group: 67 cases; POAG group: 43 cases). **C** DEG volcano plot; red represents upregulated differentially expressed genes, and green represents downregulated differentially expressed genes

After the systematic search, the datasets of the four different human microarray studies were selected for further analyses. After correcting the batch effect, we combined the four GEO datasets GSE27276, GSE2378, GSE9944 (GPL8300) and GSE9944 (GPL571) into the expression profiles of 110 samples (control group: 67 cases; POAG group: 43 cases) (Fig. 1A-B). Difference analysis was performed by the limma package. The screening conditions of different genes were *P* value < 0.05 and |logFC|> 0.585. Finally, 49 differentially expressed genes were screened, including 30 upregulated genes and 19 downregulated genes (Fig. 1C).

### Pathway analysis

**Fig. 2:**
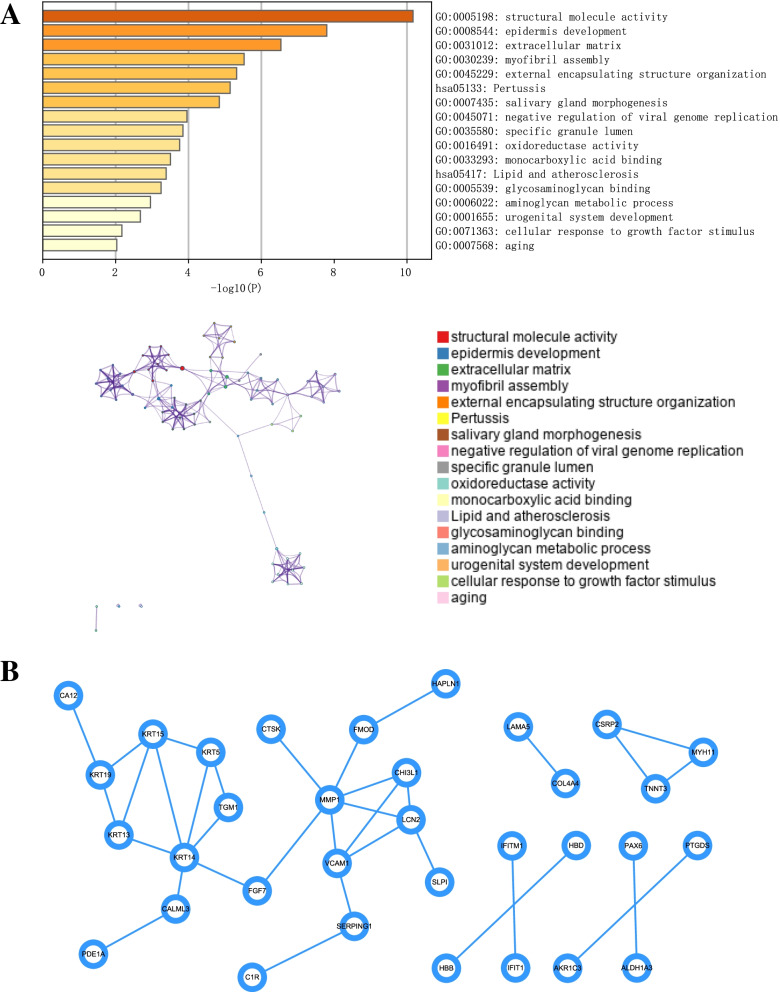
GO and PPI network analyses of DEGs. **A** GO biological function enrichment analysis. **B** PPI network analysis graph. GO, Gene Ontology; PPI, protein–protein interaction; DEGs, differentially expressed genes

We further analyzed the pathways of these 49 candidate genes in the Metascape database. The results showed that these candidate genes were mainly enriched in structural molecular activity, epidermis development, extractive matrix, oxidoreductase activity, aminoglycan metabolic process, aging and other pathways (Fig. 2A). Moreover, we analyzed the protein–protein interaction (PPI) network of genes in different gene sets by Cytoscape software (Fig. 2B).

### Key genes

**Fig. 3:**
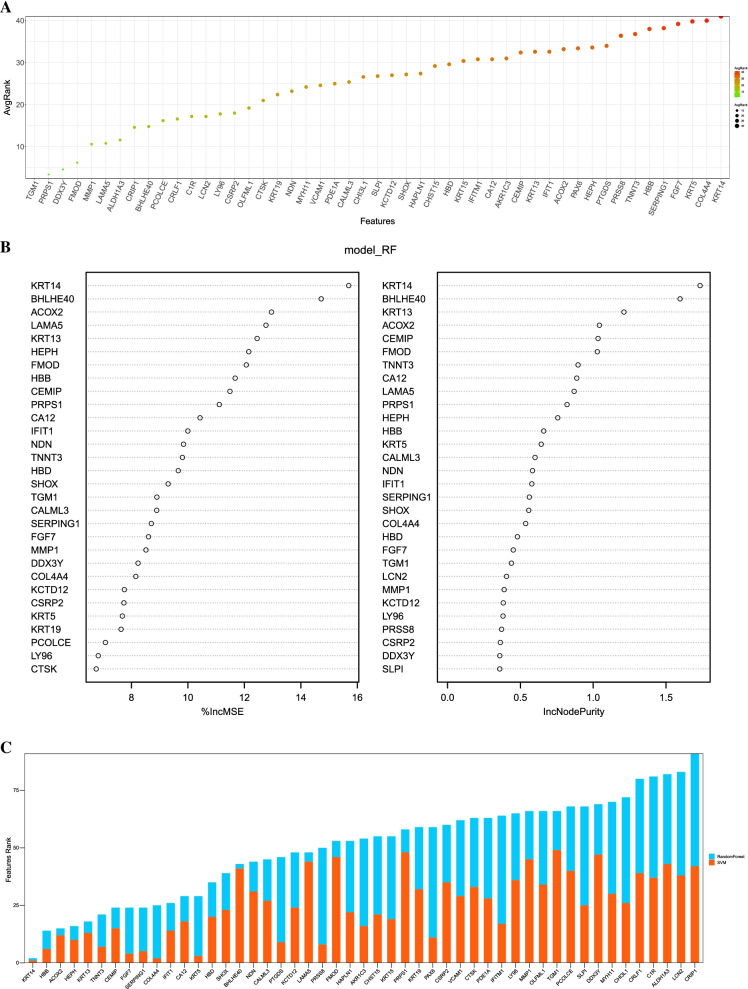
Selection of diagnostic biomarkers and identification of key genes. **A** Select POAG biomarkers by random forest. **B** Select POAG biomarkers by SVM. **C** Key genes extracted from the random forest and SVM methods. SVM, support vector machine; Keratin 14, KRT14; Hemoglobin subunit beta, HBB; Acyl-CoA Oxidase 2, ACOX2; Hephaestin, HEPH; and Keratin 13, KRT13

**Fig. 4:**
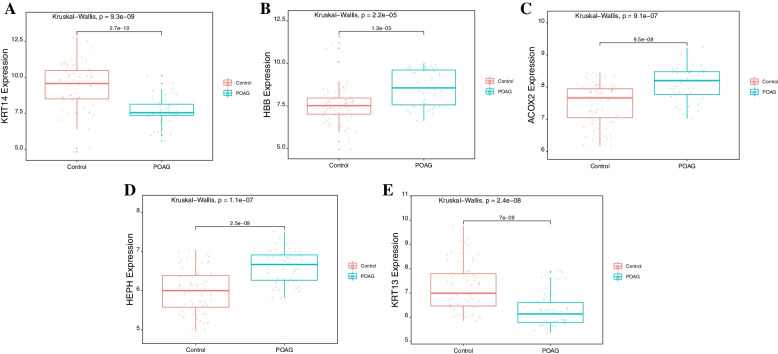
The expression of five key genes in patients with the POAG group and participants in the normal group. **A** KRT14 is downregulated in patients with POAG. **B** HBB is upregulated in patients with POAG. **C** ACOX2 is upregulated in POAG. **D** HEPH is upregulated in POAG. **E** KRT13 is downregulated in patients with POAG. *P* value < 0.05. Red represents normal groups, and green represents POAG groups. Keratin 14, KRT14; Hemoglobin subunit beta, HBB; Acyl-CoA Oxidase 2, ACOX2; Hephaestin, HEPH; and Keratin 13, KRT13

We analyzed the above 49 differentially expressed genes by random forest and SVM to screen the key genes (Fig. 3A-B). According to the comprehensive scores of the two machine learning methods, we obtained the top 5 genes as the key gene sets, which were (Keratin 14) KRT14, (Hemoglobin subunit beta) HBB, (Acyl-CoA Oxidase 2) ACOX2, (Hephaestin) HEPH and Keratin 13 (KRT13) (Fig. 3C). The expression of five key genes in the POAG group and normal group is shown in Fig. 4.

### Immune cell infiltration

**Fig. 5:**
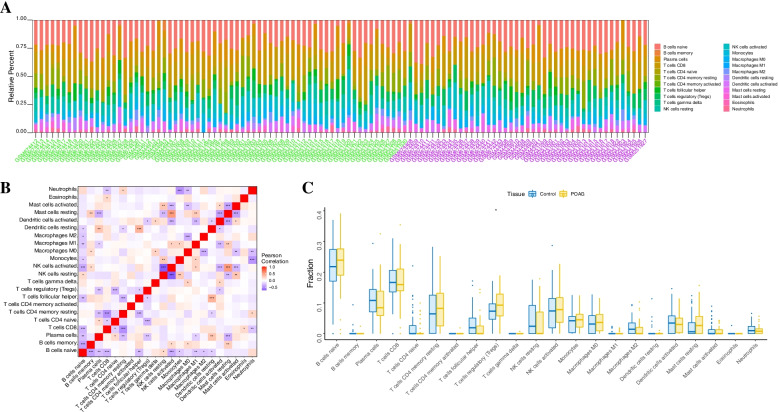
Correlation plots of immune cell infiltration analysis. **A** The proportion of 22 immune cells. **B** Correlation heatmap of 22 immune cells. Red represents a positive correlation, purple represents a negative correlation, and the darker the color is, the stronger the correlation. **C** Plot of the proportion of infiltration by 22 types of immune cells in normal control samples versus in POAG samples. Blue represents control samples; yellow represents POAG samples

The microenvironment is mainly composed of immune cells, extracellular matrix, a variety of growth factors, inflammatory factors and special physical and chemical characteristics, which significantly affect the sensitivity of disease diagnosis and clinical treatment. By analyzing the relationship between key genes and immune infiltration in the POAG dataset, we further explored the potential molecular mechanism of key genes affecting the progression of POAG. The results show that the proportion of immune cells in each patient and the correlation between immune cells are shown in Fig. 5A-B. Compared with the normal group, the T-cell regulatory (Treg) level of samples in the POAG group was significantly higher (Fig. 5C).

**Fig. 6:**
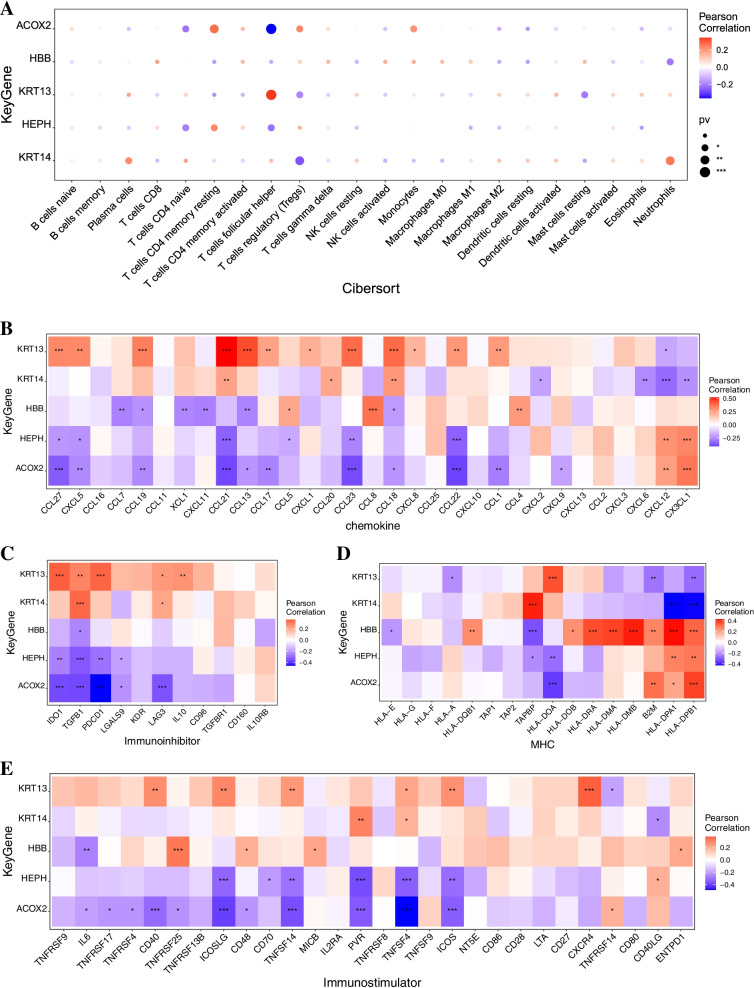
The relationship between key genes and immune cells. **A** The five key genes (KRT14, HBB, ACOX2, HEPH and KRT13) were highly correlated with immune cells. **B** The relationship between key genes and chemokines. **C** The relationship between key genes and immunoinhibitors. **D** The relationship between key genes and MHC **E** The relationship between key genes and immunostimulators. MHC, major histocompatibility complex. Keratin 14, KRT14; Hemoglobin subunit beta, HBB; Acyl-CoA Oxidase 2, ACOX2; Hephaestin, HEPH; and Keratin 13, KRT13

We further explored the relationship between key genes and immune cells. The five key genes were highly correlated with immune cells. KRT14 was positively correlated with plasma cells and neutrophils and negatively correlated with regulatory T cells (Tregs) and mast cell resetting. HBB was positively correlated with activated NK cells and monocytes and negatively correlated with resting mast cells and resting dendritic cells. ACOX2 was positively correlated with CD4 memory resting T cells and monocytes and negatively correlated with cellular helper T cells and naïve CD4 T cells. HEPH was positively correlated with memory CD4 + T-cell resetting and regulatory T cells (Tregs) and negatively correlated with naive CD4 + T cells and follicular helper T cells. KRT13 was positively correlated with follicular helper and plasma cells and negatively correlated with regulatory T cells (Tregs) and resting mast cells (Fig. 6A). We further obtained the correlation between these key genes and different immune factors from the TISIDB database, including immune modulators, chemokines and cell receptors (Fig. 6B-E). These analyses confirmed that these key genes are closely related to the level of immune cell infiltration and play an important role in the immune microenvironment.

### Key gene-related pathways

**Fig. 7:**
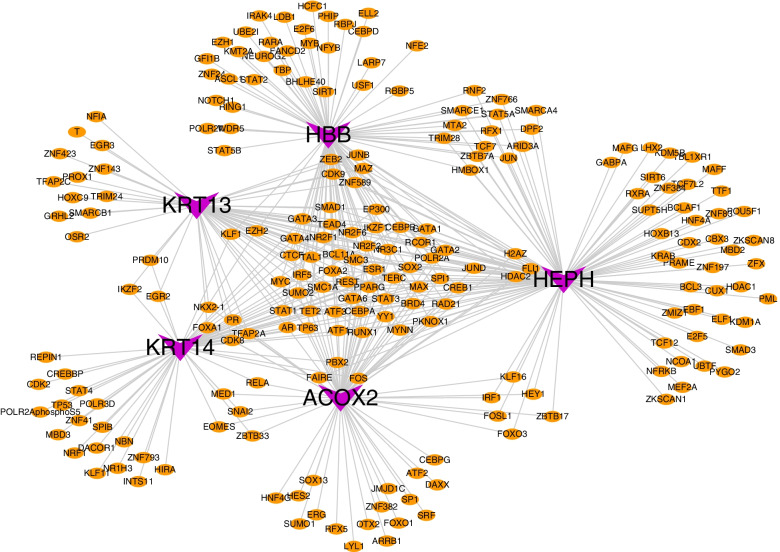
A comprehensive transcriptional regulatory network of key POAG genes (KRT14, HBB, ACOX2, HEPH and KRT13) was constructed by visualization through Cytoscape. Keratin 14, KRT14; Hemoglobin subunit beta, HBB; Acyl-CoA Oxidase 2, ACOX2; Hephaestin, HEPH; and Keratin 13, KRT13

We used these five key genes in the gene set of this analysis to further explore the transcriptional regulatory network involved in key genes. Relevant transcription factors were predicted through the Cistrome DB online database, including 55 transcription factors predicted by KRT14, 92 transcription factors predicted by HBB, 71 transcription factors predicted by ACOX2, 106 transcription factors predicted by HEPH and 57 transcription factors predicted by KRT13. Finally, a comprehensive transcriptional regulatory network of key POAG genes was constructed by visualization through Cytoscape (Fig. 7).

**Fig. 8:**
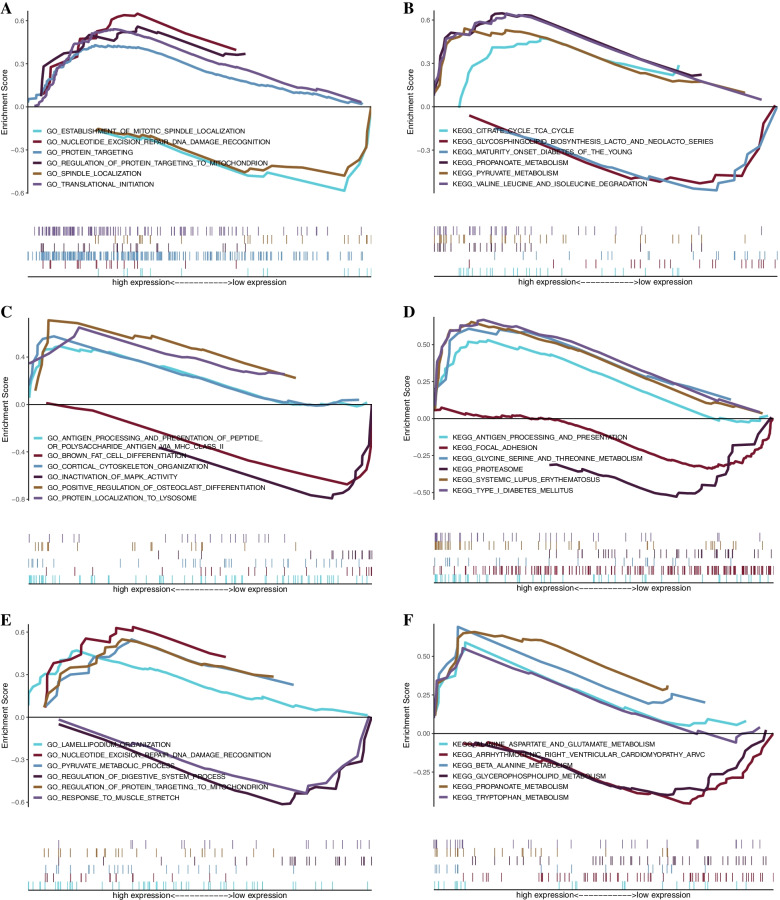
GSEA of GO and KEGG enrichment analysis for the key genes. **A-B** GSEA of GO and KEGG enrichment analysis for ACOX2. **C-D** GSEA of GO and KEGG enrichment analysis for HBB. **E–F** GSEA of GO and KEGG enrichment analyses for HEPH. GSEA, gene set enrichment analysis. Hemoglobin subunit beta, HBB; Acyl-CoA Oxidase 2, ACOX2; and Hephaestin, HEPH

**Fig. 9:**
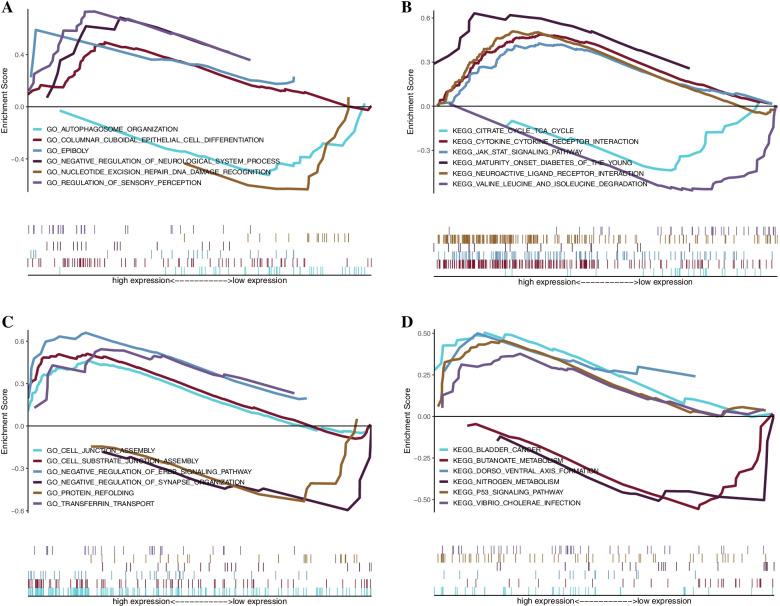
GSEA of GO and KEGG enrichment analysis for the key genes. **A-B** GSEA of GO and KEGG enrichment analysis for KRT13. **C-D** GSEA of GO and KEGG enrichment analysis for KRT14. GSEA, gene set enrichment analysis. Keratin 14, KRT14; and Keratin 13, KRT13

We studied the specific signaling pathways enriched by five key genes to explore the potential molecular mechanism of key genes affecting the progression of POAG. We selected the significantly enriched pathways shown in Figs. 8 and 9. The pathways enriched with KRT14 by GO analysis included cell substrate junction assembly, cell junction assembly and other pathways. The pathways enriched by KEGG included ladder, cancel and so on Butanoate metabolism and other channels [17]. The pathways enriched with HBB by GO analysis included brown fat cell differentiation and corporate cytoskeleton organization. The pathways enriched by KEGG include Angel processing and presentation and focal adhesion. The pathways enriched with ACOX2 by GO analysis included spindle localization and transitional initiation. The pathways enriched by KEGG include promote metabolism and pyruvate metabolism. The pathways enriched with HEPH by GO analysis included numeric expression repair DNA recognition and lamellipodium organization. The pathways enriched by KEGG included glycerophospholipid metabolism and beta alanine metabolism. The pathways enriched with KRT13 by GO analysis included autophagosome organization and column cuboidal epithelial cell differentiation. The pathways enriched by KEGG included the circuit cycle, TCA cycle, and cytokine receptor interaction (Fig. 9).

### Gene regulatory network analysis of key genes in POAG

**Fig. 10:**
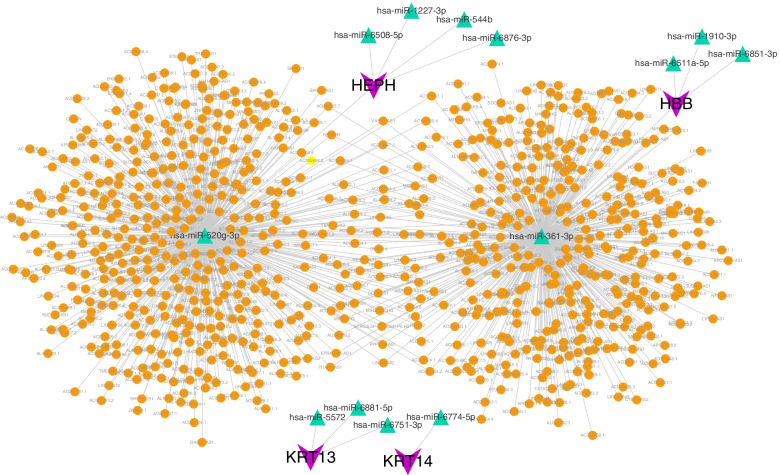
The five key genes through the miRWalk database and ENCORI database to obtain their possible miRNAs and lncRNAs. Keratin 14, KRT14; Hemoglobin subunit beta, HBB; Acyl-CoA Oxidase 2, ACOX2; Hephaestin, HEPH; and Keratin 13, KRT13

We predicted and analyzed the five key genes through the miRWalk database and ENCORI database to obtain their possible miRNAs and lncRNAs. First, the mRNA–miRNA relationship pairs related to these five key mRNAs were extracted from the miRWalk database. We retained only 35 mRNA–miRNA relationship pairs with TargetScan of 1 or miRDB of 1 (including 4 mRNAs and 13 miRNAs). Then, we predicted the interacting lncRNAs according to these miRNAs, in which 1112 pairs of interactions (including 2 miRNAs and 823 lncRNAs) were predicted. Finally, we constructed the ceRNA network through Cytoscape (V3.7) (Fig. 10).

### POAG biomarkers

**Fig. 11:**
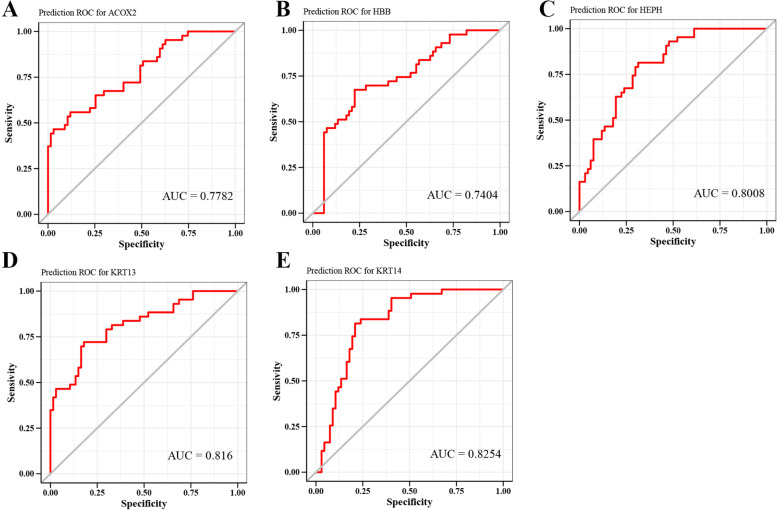
Verification of biomarkers. **A** Prediction ROC for ACOX2. **B** Prediction ROC for HBB. **C** Prediction ROC for HEPH. **D** Prediction ROC for KRT13. **E** Prediction ROC for KRT14. Keratin 14, KRT14; Hemoglobin subunit beta, HBB; Acyl-CoA Oxidase 2, ACOX2; Hephaestin, HEPH; and Keratin 13, KRT13

We discussed the prediction efficiency of key genes through the ROC curve verified by diagnostic efficiency. The results showed that the area under the AUC of KRT14 was 0.825; the area under the AUC of HBB was 0.740; the area under the AUC of ACOX2 was 0.778; the area under the AUC of HEPH was 0.801; and the area under the AUC of KRT13 was 0.816. These results show that these five key genes have good prediction efficiency for POAG and may better predict the occurrence and development of diseases (Fig. 11A-E).

### Drug targeting prediction in POAG

**Fig. 12:**
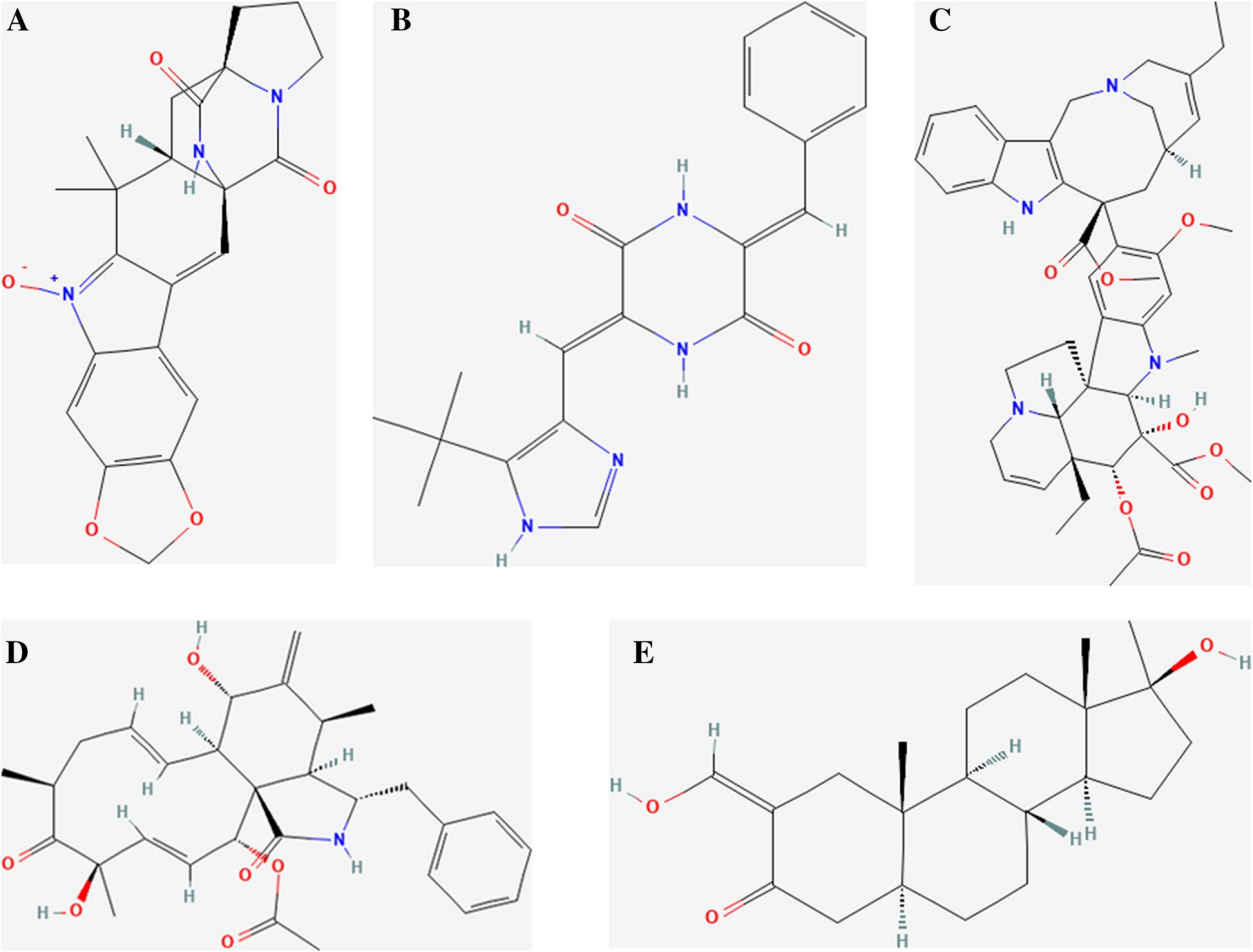
Drug targeting prediction in POAG. **A** Avrainvillamide-analysis-3. **B** NPI-2358. **C** vinorelbine. **D** cytochalasin-D. **E** oxymethylone

We divided the differentially expressed mRNAs into upregulated and downregulated groups and predicted the drug targets of the differentially expressed genes through the Connectivity Map database. The results showed that the expression profiles of drug disturbances, such as avrainvillamide-analysis-3, cytochalasin-D, NPI-2358, oxymethylone and vinorelbine, were negatively correlated with the expression profiles of disease disturbances (Fig. 12). This finding indicated that these drugs may reduce or even reverse the POAG disease state.

## Discussion

POAG is a chronic retinal neurodegeneration disease characterized by changes in the anterior and posterior segments of the eye; in addition, serious damage may be detected in the TM and ONH [1, 5, 8]. Lowering IOP using drugs or surgery is the only intervention currently available [6]. However, clinical evidence indicates that lowering IOP does not prevent progression in all POAG patients. Consequently, non-IOP factors are involved in the disease [2]. With the rapid development of science and technology, bioinformatics provides a powerful strategy for the screening of molecular markers [18]. Biomarkers reflect changes at the molecular level and can accurately monitor pathological changes in the TM and ONH and provide important information for the diagnosis of POAG [5, 15, 19–23]. Nevertheless, the detection of POAG lesions using molecular biology methods is suboptimal, and treatments are currently in a limited pharmacotherapy phase [6].

In the present study, to screen the pathogenic genes involved in POAG, an integrated analysis was performed by using microarray datasets in glaucoma derived from the GEO database. The functional annotation and potential pathways of DEGs were additionally examined by GO and KEGG enrichment analyses. A POAG-specific transcriptional regulatory network was constructed to identify crucial transcription factors that target the key genes in patients with POAG. We used random forest and the SVM method to screen five potential key genes in both human TM and ONH tissue. A growing number of researchers realize that immune infiltration is related to the diagnosis of POAG [10–12]. Therefore, analyzing the pattern of POAG immune cell infiltration and finding specific diagnostic markers have profound significance for POAG patients. Subsequently, the CIBERSORT algorithm performed deconvolution analysis on the immune microenvironment to assess the proportion of immune cells in POAG.

We identified key differentially expressed genes between POAG and normal tissues by performing a combined analysis of TM and ONH. A total of 49 DEGs were identified, including 19 downregulated genes and 30 upregulated genes. These 49 differentially expressed genes were further analyzed by random forest and SVM to screen the key genes. Five genes (KRT14, HBB, ACOX2, HEPH and KRT13) were significantly changed. We found that these key genes were highly correlated with immune cells. The immune microenvironment is composed of a variety of lymphocytes, such as T cells, B cells and macrophages, etc. [24]. From the immune infiltration analysis, we found that there was a significant difference in the relative cell content of 22 types of immune cells (e.g., B cells naïve, B cell memory, plasma cells, T cells CD8, T cells CD4 naïve, T cells CD4 memory resting) in normal control samples versus in POAG samples.

KRT14 is a member of the type I keratin family of intermediate filament proteins [25]. KRT 14 is expressed in a variety of cells in humans. It is recovered as a heterodimer with KRT5 and forms the cytoskeleton of epithelial cells [26, 27]. Gautam et al., using multispecies single-cell transcriptomic analysis of the human eye, found that KRT 14 was expressed in corneal epithelial cells [28]. However, previous studies did not perform a deeper investigation of POAG. In our study, we found that KRT14 was expressed in both TM and ON tissues in humans. KRT14 was highly correlated with immune cells (plasma cells, neutrophils, etc., and negatively correlated with regulatory T cells (Tregs) and mast cell resetting. HBB (hemoglobin subunit beta) is encoded by the HBB gene on human chromosome 11 [20]. HBB was positively correlated with activated NK cells and monocytes and negatively correlated with resting mast cells and resting dendritic cells. The ACOX2 gene encodes the enzyme Acyl-CoA oxidase 2 in human autosome 3, oxidizing the Coenzyme A esters of bile acid and di-trihydroxycholestanoic acid intermediates. It is located in the peroxisomes of cells and has a tripeptide at the C-terminal end of the protein formed by serine-lysine-leucine that serves as a peroxisome localization signal. Deficiency of this enzyme causes an accumulation of fatty acids and bile intermediates, generating Zellweger syndrome. ACOX2 was positively correlated with CD4 memory resting T cells and monocytes and negatively correlated with cellular helper T cells and naïve CD4 T cells. HEPH (Hephaestin) is involved in the metabolism and homeostasis of iron and possibly copper [29]. It is a transmembrane copper-dependent ferroxidase responsible for transporting dietary iron from intestinal enterocytes into the circulatory system. HEPH was positively correlated with memory CD4 + T-cell resetting and regulatory T cells (Tregs) and negatively correlated with naive CD4 + T cells and follicular helper T cells. The KRT13 (keratin 13) gene encodes a type I cytokeratin that is expressed in the differentiated cells of noncornified stratified squamous epithelia [30, 31]. KRT13 is positively correlated with T-cell follicular helper and plasma cells and negatively correlated with T-cell regulatory (Tregs) and resting mast cells.

The ocular innate immune response relevant to glaucoma involves the complement cascade, microglia, astrocytes, and Müller cells. Autoantibodies, tumor necrosis factor (TNF)-alpha, and other products of adaptive B and T cells are also featured prominently in the glaucomatous adaptive immune response. However, research has not provided clear immune infiltration among these cells [9]. Thus, in our study, based on the immune infiltration analysis between key genes and immune cells, we divided the differentially expressed mRNAs into upregulated and downregulated groups and predicted the drug targets of the differentially expressed genes through the Connectivity Map database. The results showed that the expression profiles of drug disturbances such as avrainvillamide-analysis-3, cytochalasin-D, NPI-2358, oxymethylone and vinorelbine were negatively correlated with the expression profiles of disease disturbances. NPI-2358 and vinorelbine are tubulin inhibitors. Cytochalasin-d is an actin polymerization inhibitor. Oxymetholone is an androgen receptor agonist. avrainvillamide-analog-3is a nucleophosmin inhibitor. This finding indicated that these drugs may reduce or even reverse the POAG disease state.

There were several limitations to this study. First, due to the limited number of samples, there is still a need to confirm these preclinical observations in future clinical studies for novel biomarkers. Second, CIBERSORT is based on the principle of linear support vector regression and uses gene expression data in reverse to deduce the result of immune cell infiltration. Indeed, it is not based on experimental data, and further verification of immune cell infiltration by a large number of experiments is needed. Third, we performed mining and analysis of previously published data; although some previous studies showed similar results, the related molecules and their mechanisms at the molecular, cell, and tissue levels require validation.

In conclusion, the overlap of the random forest and SVM algorithms was obtained, and five key genes were eventually recognized in both human TM and ONH tissue. We found that it may be used as a diagnostic marker for POAG. To understand POAG development, GO and GSEA of the selected genes supplied a more specific molecular mechanism. To date, the relationship between key genes and immune infiltration in both TM and ONH tissues has been rarely reported. The mechanism of key genes and immune infiltration-related factors in the diagnosis of POAG remains to be explored. Further investigation of these immune cells may identify targets of immunotherapy for POAG and help POAG patients benefit from immunomodulatory therapy.

## Materials and methods

### Material and data

The datasets used in the present study were downloaded from the National Center of Biotechnology Information (NCBI) Gene Expression Omnibus (GEO). Four sets of POAG mRNA (GSE27276-GPL2507, GSE2378-GPL8300, GSE9944-GPL8300, GSE9944-GPL571) data were obtained. The detailed information is shown in Table 1. In total, 43 POAG patients and 67 normal individuals were enrolled in our study from these four datasets.

**Table 1:** Characteristics of four datasets of POAG mRNA data

Series matrix file data	Annotation file	Number of participants in the POAG group	Number of participants in the normal group	Sample type
GSE27276	GPL2507	19	17	Trabecular meshwork
GSE2378	GPL8300	7	6	Optic nerve head
GSE9944	GPL8300	13	6	Optic nerve head
GSE9944	GPL571	6	36	Optic nerve head

### Screening of key genes

The differentially expressed genes (DEGs) in the GEO dataset (*P* < 0.05 and |logFC|> 0.585) were screened by difference analysis, and then the candidate gene sets were further screened by random forest and SVM algorithms. Among them, random forest is an integrated learning algorithm based on a decision tree. Multiple samples are selected from the sample sets as a training set by sampling with replacement. The decision tree is generated from the obtained sample sets by sampling. At each generated node, features without repetition are randomly selected. Based on optimal features to divide the sample sets, the prediction results are determined. In this study, the features were evaluated by the random forest algorithm. Using %IncMSE to evaluate the significance, 1000 trees were built and repeated 50 times. SVM is a machine learning method based on SVM. Optimal variables were identified by deleting SVM-generated feature vectors. For the sake of further recognizing the diagnostic value of disease biomarkers, a support vector machine model was established according to the “e1071” software package. Finally, the top features were retained for follow-up analysis.

### GO and KEGG functional annotation

The differentially expressed genes were functionally annotated using the Metascape database (www.metascape.org) to comprehensively explore the functional correlation of these genes. For the specific genes, we performed Gene Ontology (GO) analysis and Kyoto Encyclopedia of Gene Genome (KEGG) pathway analysis. Minimum overlap ≥ 3 and P ≤ 0.01 were considered statistically significant.

### Analysis of immune cell infiltration

CIBERSORT is widely used to evaluate the type of immune cell in the microenvironment. The tool is based on the principle of a linear support vector to perform deconvolution analysis on the expression matrix of immune cell subtypes. It contains 547 biomarkers and 22 phenotypes of human immune cells, covering plasma cells, B cells, T cells, and myeloid cell subsets. Using the CIBERSORT algorithm, this study analyzed data from POAG patients and quantified the relative proportions of 22 infiltrating immune cells. Furthermore, this study performed Pearson correlation analysis on immune cells and gene expression.

### Gene regulatory network analysis of key genes

The Cistrome DB is an up-to-date, scalable, and powerful tool to process large batches of ChIP-seq and DNase-seq datasets, which map the genome-wide locations of transcription factor-binding sites, histone posttranslational modifications and regions of chromatin accessible to endonuclease activity [32]. Currently, the Cistrome DB contains approximately 30,451 human and 26,013 mouse samples. This study explored the regulatory relationships between transcription factors and key genes based on the Cistrome DB database with genome file setting as hg38 and the transcription initiation site 10 KB, which was visualized through Cytoscape.

### Gene set enrichment analysis (GSEA)

GSEA uses a predefined gene set to rank the genes according to the degree of differential expression in the two types of samples and then tests whether the preset gene set is enriched at the top ranking or bottom ranking. This study compared the differences in KEGG and GO signaling pathways between participants in the high expression group and the low expression group by GSEA to explore the molecular mechanism of key genes in the two groups, in which the number of replacements was set to 1000 and the type of replacement was set to phenotype.

### Drug targeting prediction

The connectivity map (CMAP), which was developed by the Broad Institute, is a resource created to enable data-driven studies on drug mode-of-action and drug repositioning; it is mainly used to reveal the functional relationship among small molecular compounds, genes and disease status. It contains the microarray data of 1309 small molecule drugs before and after treatment of 5 human disease cell lines. It provides various treatment conditions, including different drugs, different concentrations, different treatment durations and so on.

### Statistical analysis

All statistical analyses were performed in R language (version 3.6). All statistical tests were bilateral (*P* < 0.05).

## Data Availability

The selected datasets generated and/or analyzed during the current study are available in the Gene Expression Omnibus (GEO) repository (https://www.ncbi.nlm.nih.gov/geo/query/acc.cgi?acc=GSE27276; https://www.ncbi.nlm.nih.gov/geo/query/acc.cgi?acc=GSE2378; https://www.ncbi.nlm.nih.gov/geo/query/acc.cgi?acc=GSE9944). Based on these datasets, our data generated or analyzed during this study are available from the corresponding author on reasonable request.
